# Agreement Between Two Nutritional Assessment Scores as Markers of Malnutrition in Patients with End-stage Renal Disease

**DOI:** 10.7759/cureus.7429

**Published:** 2020-03-26

**Authors:** Abdul Rehman Arshad, Shanzay Jamal, Khadija Amanullah

**Affiliations:** 1 Nephrology, Combined Military Hospital Peshawar, Peshawar, PAK; 2 Nephrology, Pak-Emirates Military Hospital, Rawalpindi, PAK

**Keywords:** hemodialyis, nutrition, end stage renal disease, malnutrition

## Abstract

Background

Malnutrition is directly related to morbidity and mortality in end-stage renal disease. This should be picked up using simple techniques.

Methods

Adult patients on maintenance haemodialysis were included using a consecutive sampling technique. Compliance was assessed from attendance register (minimum 75% attendance for good compliance). Hypoalbuminemia signified malnutrition. Blood samples for measurement of haemoglobin, serum albumin, calcium and phosphate levels were drawn from the dialyser tubing at the start of the first of the two haemodialysis sessions for each patient. Height and weight were recorded at the end of the first haemodialysis session for each patient. Mini Nutritional Assessment Questionnaire and Council on Nutrition Appetite Questionnaire were administered in direct face-to-face interviews during two consecutive dialysis sessions.

Results

There were 116 patients aged 53.46± 14.39 years. Majority were males (83.6%) and on twice a week haemodialysis (69.0%). Malnutrition was present in 30 (25.9%) patients. Serum albumin had a significant relationship with both haemoglobin (R = 0.399; *p* < 0.001) and serum phosphate levels (R = 0.253; *p *= 0.006) but not body mass index (R = 0.028; *p* = 0.769). Mean Mini Nutritional Assessment and Council on Nutrition Appetite scores were 19.45± 5.10 and 26.76± 6.28, respectively. Based on Mini Nutritional Assessment scores, 31 (26.7%) patients were malnourished, 59 (50.9%) were at risk of malnutrition, and 26 (22.4%) had normal nutritional status. Council on Nutrition Appetite scores were low in 65 (56.0%) patients, indicating risk of weight loss in next six months. Serum albumin had significant correlation with Mini Nutritional Assessment scores (R = 0.381; *p* < 0.001) and Council on Nutrition Appetite scores (R = 0.290; *p *= 0.002). Slopes of linear regression for Mini Nutritional Assessment and Council on Nutrition Appetite scores were not statistically different (*p *= 0.202).

Conclusions

Mini Nutritional Assessment and Council on Nutrition Appetite scores had a similar correlation with serum albumin levels. Either of the two could be used for evaluation of malnutrition in end-stage renal disease.

## Introduction

The world population is ageing rapidly. A proportion of people aged ≥65 years is expected to double from 8% in 2010 to 16% by 2050 [1]. The burden of end-stage renal disease would also increase exponentially [2]. Malnutrition is an important complication in such cases. The main contributing factors could be uraemia and metabolic acidosis causing anorexia, nausea, and vomiting. In addition, dietary restrictions, recurrent infections as well as protein loss during haemodialysis (HD) sessions contribute significantly. Malnutrition affects 18-75% patients on long-term HD [3]. Assessment and management of malnutrition remain an important and integral part of management in patients undergoing maintenance HD. Yet, this aspect is frequently ignored in daily clinical practice. Considering the association of malnutrition with mortality in these patients, it is absolutely essential to periodically assess their nutritional status and institute measures for treatment in a timely manner. Provision of adequate nutrition to HD patients has already been shown to be associated with better clinical outcomes [4].

There is no single gold standard test or method for the diagnosis of malnutrition [5]. Mostly, the assessment takes into account multiple clinical features and laboratory investigations that together provide an overall picture of someone’s nutritional status. Being labour intensive and time consuming, this becomes too cumbersome for routine use. Numerous scoring systems are already available. Most of these like Subjective Global Assessment, Short Nutritional Assessment Questionnaire, and Malnutrition Universal Screening Tool were devised for use in general population, whereas a few others (such as Geriatric Nutritional Risk Index) have been validated for use in adult HD patients. It is important to realize that each of these questionnaires has its own limitations and thus not suited for universal use.

The objective of this study was to find a questionnaire suitable for use in our HD patients. After extensive literature review and considering pros and cons, we shortlisted two questionnaires. We took into account their contents and ease of administration as well as the time required to gather all relevant information. They were Mini Nutritional Assessment Questionnaire (MNA) and Council on Nutrition Appetite Questionnaire (CNA). This study was carried out to see if anyone of these performed better in picking up malnutrition. This would, in turn, help us choose the best one for implementation as a preferred tool in our dialysis centre.

## Materials and methods

This cross-sectional study was carried out at the Department of Nephrology, Pak Emirates Military Hospital Rawalpindi from Feb to April 2019. It was approved by the Ethics Review Committee of the hospital. Adult patients with end-stage renal disease on maintenance HD were enrolled from the dialysis unit after obtaining informed consent. A consecutive sampling technique was employed. We excluded patients on HD for less than one month, those with poor compliance to HD, hospital admission during the last 30 days (irrespective of the reason and duration of admission) and unwilling patients. Compliance was assessed using attendance register maintained at the dialysis unit, with minimum 75% attendance during the last four weeks deemed satisfactory. Serum albumin was taken as a surrogate marker of nutritional status amongst the patients, with hypoalbuminemia (serum albumin <35 g/L) reflecting malnutrition. Blood samples for measurement of haemoglobin and serum albumin, calcium, and phosphate levels were drawn from the dialyser tubing at the start of the first of the two HD sessions for each patient. Mini Nutritional Assessment Questionnaire and Council on Nutrition Appetite Questionnaire were administered to all patients by two different doctors in direct face-to-face interviews during two consecutive dialysis sessions. Height and weight were recorded at the end of the first HD session for each patient. Data was analysed using SPSS version 20. Linear regression was used to determine the correlation of the two nutritional assessment scores with serum albumin levels. The two regression slopes were compared using Free Statistics Calculator [6].

## Results

There were 116 patients included in this study, whose baseline characteristics are shown in Table 1.

**Table 1:** Baseline characteristics HD: haemodialysis; BMI: body mass index

Parameter		Value
Age (years)		53.46 ± 14.39
Gender distribution	Males	97 (83.6%)
	Females	19 (16.4%)
Frequency of HD	Twice a week	80 (69.0%)
	Thrice a week	36 (31.0%)
Duration on HD (months)		8 (interquartile range: 3-24)
BMI (kg/m^2^)		23.59 ± 4.51
Haemoglobin (g/dL)		9.80 ± 1.71
Serum albumin (g/L)		38.54 ± 7.64
Serum phosphate (mmol/L)		0.76 ± 0.69

**Figure 1:**
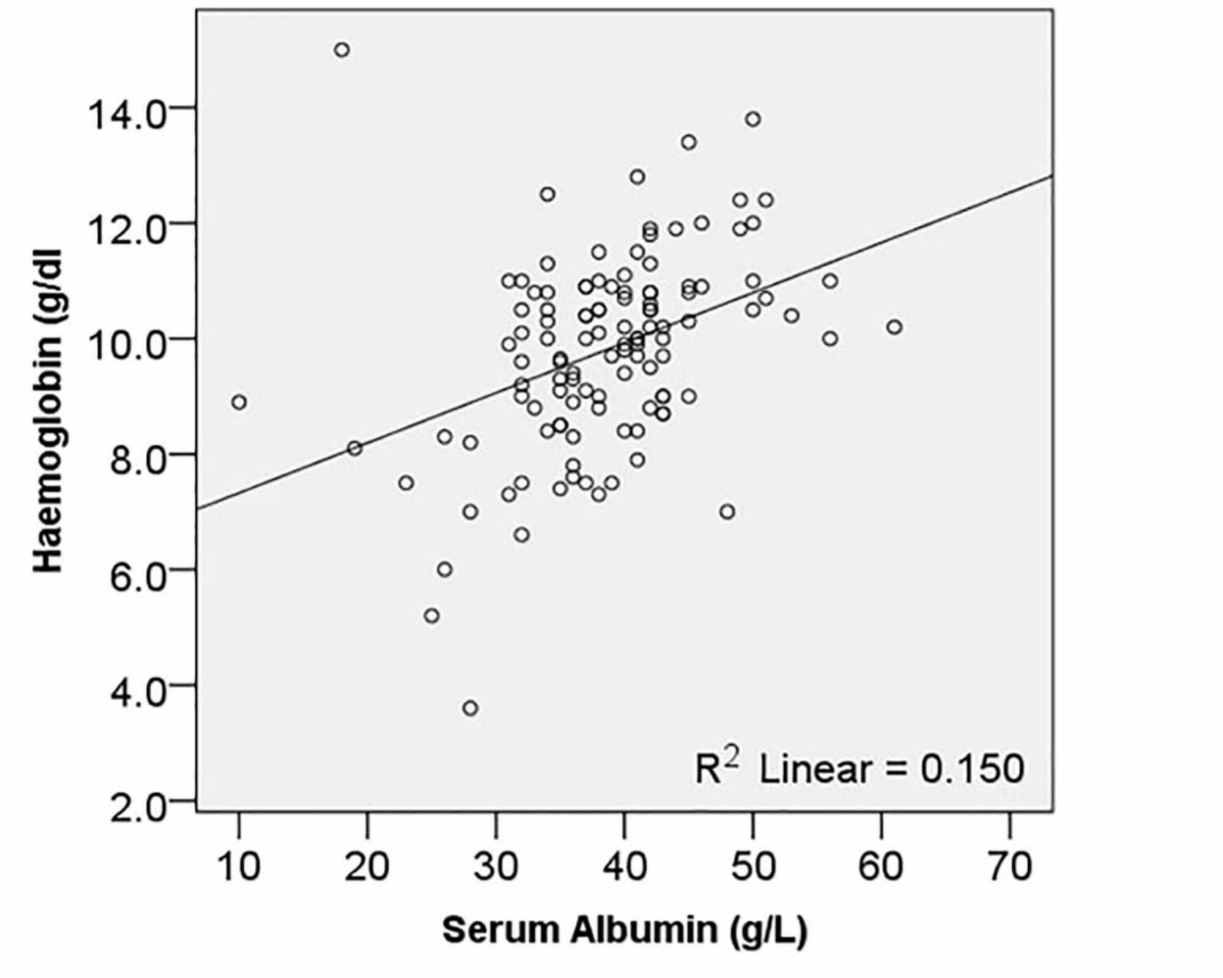
Relationship of serum albumin with haemoglobin levels

**Figure 2:**
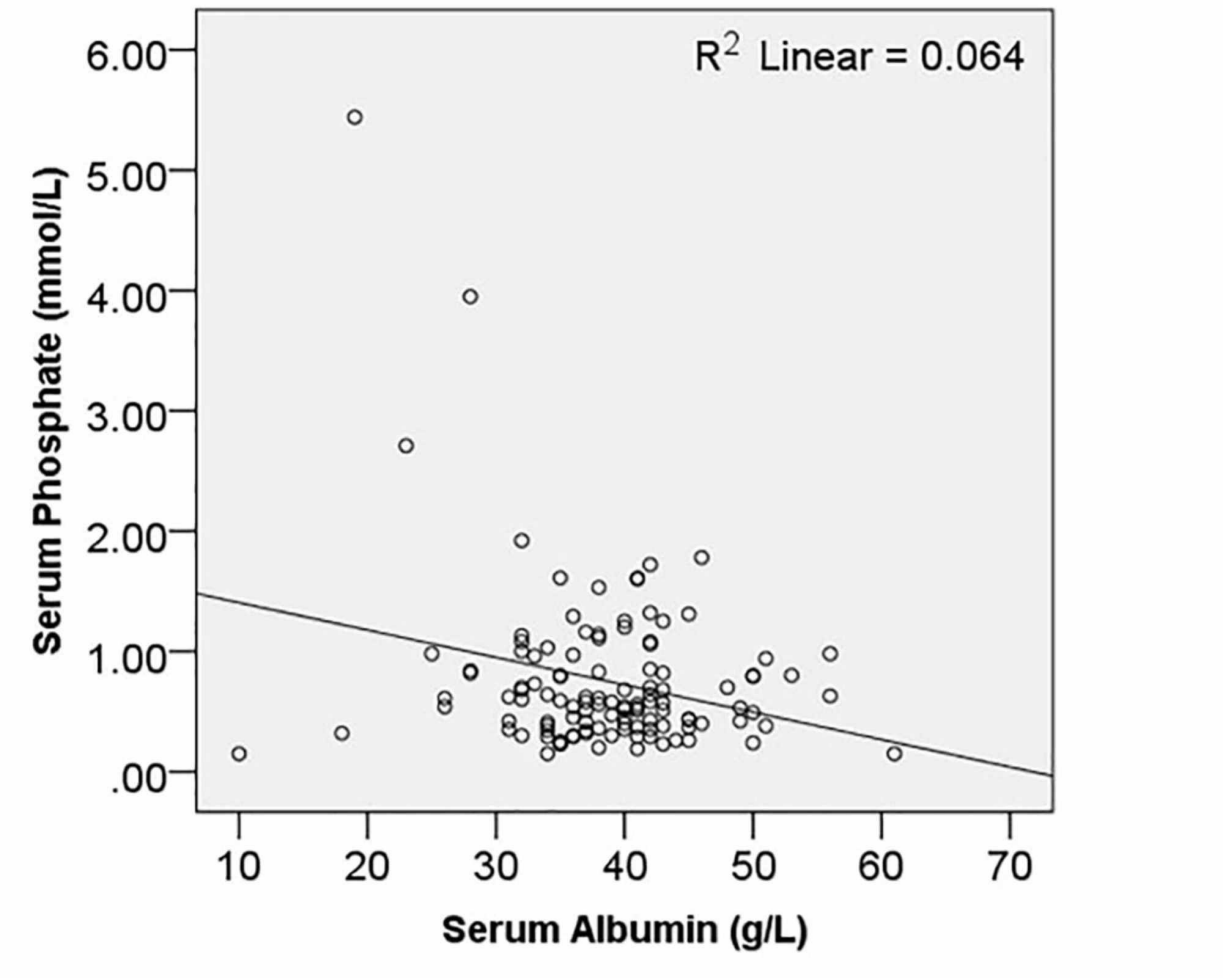
Relationship of serum albumin with phosphate levels

Nearly one fourth patients (30; 25.9%) had hypoalbuminemia. Serum albumin had a significant relationship with both haemoglobin (R = 0.399; *p* < 0.001) and serum phosphate levels (R = 0.253; *p *= 0.006), as shown in Figures 1 and 2. However, the correlation with body mass index (BMI) was not statistically significant (R = 0.028; *p* = 0.769).

**Figure 3:**
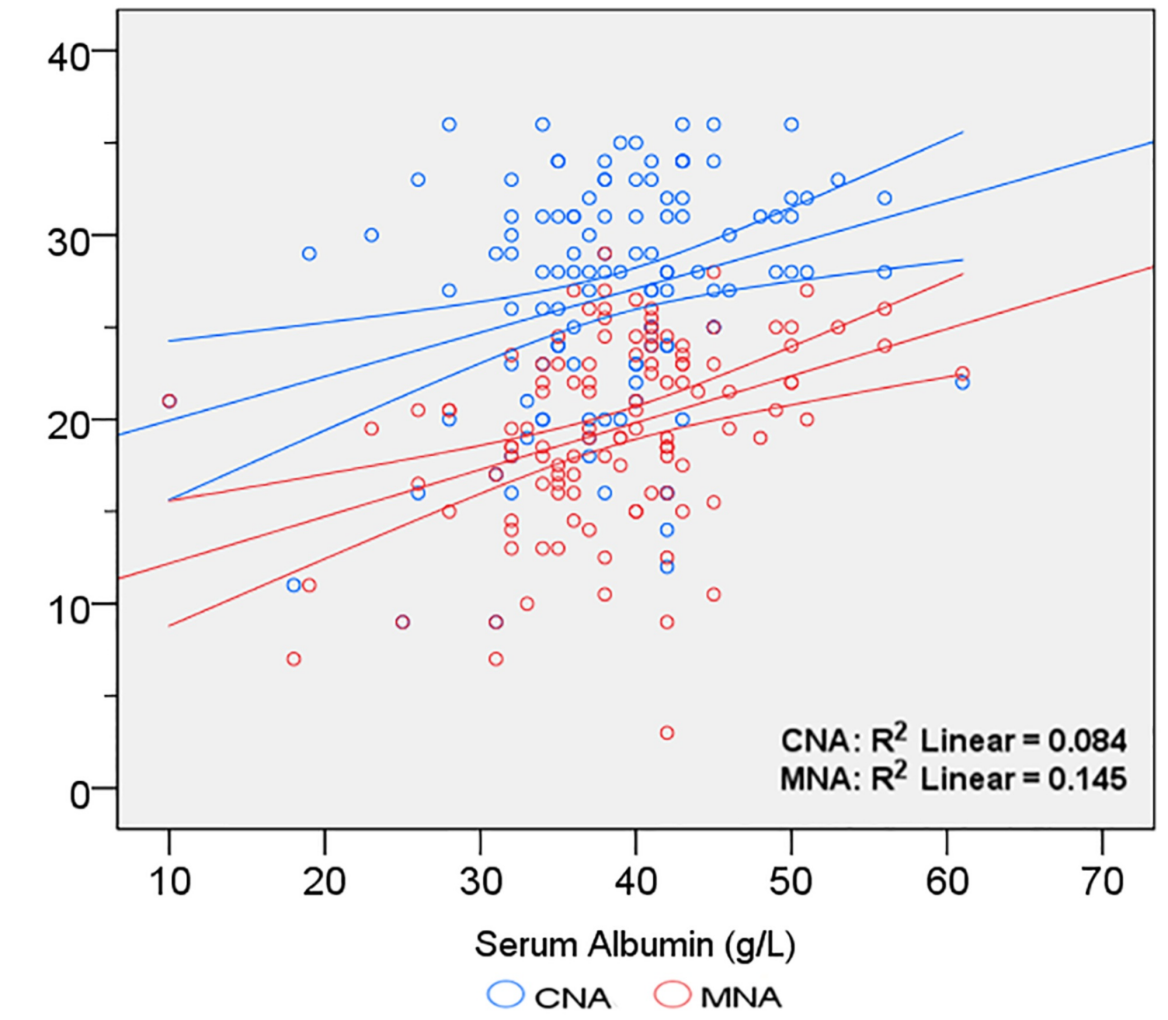
Relationship of serum albumin with MNA and CNA scores Curved lines show 95% confidence intervals for the mean. MNA: Mini Nutritional Assessment Questionnaire; CNA: Council on Nutrition Appetite Questionnaire

Mean MNA score was 19.45± 5.10. This was not different amongst males and females (19.74 ± 5.11 and 17.97± 4.96; *p *= 0.169). Amongst our patients, 31 (26.7%) were malnourished, 59 (50.9%) were at risk of malnutrition, and 26 (22.4%) had normal nutritional status. Linear regression analysis revealed significant correlation between MNA scores and serum albumin (R = 0.381; *p *< 0.001). Mean CNA score was 26.76 ± 6.28. This was lower in males as compared to females (19.74 ± 5.97 and 23.05 ± 6.65; *p *= 0.013). In 65 (56.0%) patients, CNA scores were ≤28, indicating significant risk of weight loss greater than 5% during the next six months. Linear regression analysis revealed significant correlation between CNA scores and serum albumin (R = 0.290; *p *= 0.002). The slope of the linear regression line between serum albumin and MNA was 0.570, with standard error of 0.130. The slope of the linear regression line between serum albumin and CNA was 0.353, with standard error of 0.109. This difference was not statistically significant (*p *= 0.202). Results of linear regression analysis are depicted in Figure 3.

## Discussion

Despite the strong link between malnutrition and morbidity/ mortality in end-stage renal disease, the gold standard for assessment of malnutrition is still not clear [7]. Subjective Global Assessment (SGA) and its modification, the Malnutrition- Inflammation Score, are generally regarded as the most commonly used tools for evaluation of the nutritional status of HD patients [8]. Their use in resource-limited settings is not practical, especially considering the need to train staff for effective use. Keeping this in mind, we sought simpler tools that could be used with minimal training. MNA and CNA were the best answers under these circumstances.

MNA is a time-tested and well-validated nutritional status screening tool. It is available in two forms. The full questionnaire consists of 18 questions split into screening and assessment components and evaluates nutritional status in four areas (basic anthropometrics, dietary intake, global indicators, and self-assessed health status). MNA-Short Form comprises only six questions from the screening section of the full questionnaire. It has the same validity and accuracy as the full questionnaire and makes administration in busy clinics and dialysis centres simpler [9]. MNA has a sensitivity comparable to SGA for the diagnosis of malnutrition [10]. Limited evidence has demonstrated its usefulness as a marker of nutritional status in the elderly (>60 years) HD patients as well [11].

CNA, on the other hand, comprises eight questions to assess appetite. It is suitable for self-administration and does not require specific input from the treating team. This has primarily been used for the evaluation of appetite in elderly nursing home residents. Despite an extensive literature search on PubMed, we could not identify any study using this for assessment of nutritional status in HD patients. This is a major strength of this study. We have demonstrated the utility of this infrequently used questionnaire as an effective tool for picking up malnutrition in our patients.

Malnutrition affected a significant proportion of our patients. Agboton, *et al*. documented adequate nutritional status in a little more than half of their West African patients [12]. Similarly, nearly 70% of HD-dependent patients were reported to have a good nutritional status in a study from Poland [13]. Our statistics are difficult to compare with other studies because of differences in demographic characteristics such as age, gender distribution, and HD vintage.

Serum albumin was chosen as a surrogate marker of malnutrition in this study because hypoalbuminemia is the single most important marker of malnutrition and mortality in dialysis patients [14]. In addition, it is widely available and very practical in differentiating between good and poor nutritional status due to a clearly defined cut-off value. Our results have demonstrated that MNA is a reliable marker of nutritional status in younger patients as well. Both MNA and CNA were equally good at predicting hypoalbuminemia in our patients. Though either of the two could be used, there is an argument for favouring CAN since it is completely self-administrable and does not need even the little input from the healthcare workers that the MNA demands.

There was no relationship between BMI and serum albumin levels in our patients. This is in keeping with the results of another study done at our centre almost 13 years ago [15]. BMI is limited in its ability to assess changes in body composition, specifically the relationship between fats and muscles, as well as the changes that occur with fluid overload without any change in lean body mass [16]. BMI is thus not a suitable marker of malnutrition in HD patients. This has been demonstrated in several studies done previously [17].

Significant temporal variations in body weight are a common phenomenon amongst HD patients. This highlights an important limitation of MNA, whereby the inclusion of BMI, having a weightage of 10% in the total score, makes evaluation of HD patients tricky. A study done on 95 patients in Taiwan concluded that modification of MNA by deleting the BMI question and reallocating its marks to other anthropometric measurements was more accurate in predicting the nutritional status of HD patients as compared to the original full version of MNA [18].

Not all of our patients were on thrice-a-week HD schedule. Similar trends have been reported previously as well, both locally as well as in other countries [19-20]. Though the frequency of HD does affect nutritional status, this was not a concern in our patients since both the questionnaires were administered to every single patient included in this study. 

The major strength of this study is the comparison of an infrequently used questionnaire with one that has previously been validated in this subgroup of patients. The results would open new avenues in trouble-free evaluation of the nutritional status of HD patients. We suggest that more studies should be carried out using CNA in other cohorts of dialysis-dependent populations, so as to validate our results. The major limitation of this study is the failure to measure C-reactive protein levels in our patients. Thus, we cannot comment whether the low albumin levels in one-fourth of the patients truly reflected poor nutritional status or rather indicated heightened chronic inflammatory response to HD.

## Conclusions

Based on the main findings of this study, we can conclude that malnutrition is a significant problem in our HD patients. Both the MNA and CAN scores have a significant correlation with serum albumin levels in such patients and are equally good in predicting hypoalbuminemia and malnutrition. CNA has an edge over MNA because, in our experience, it is easier to use and does not put any burden on the healthcare resources.

## Appendices

Part of this data was presented as a poster at 2nd Young Scientists Research Conference organized by Foundation University Islamabad (Pakistan) in July 2019.
